# Seed germination of *Caragana* species from different regions is strongly driven by environmental cues and not phylogenetic signals

**DOI:** 10.1038/s41598-017-11294-x

**Published:** 2017-09-12

**Authors:** Xiang-Wen Fang, Juan-Juan Zhang, Dang-Hui Xu, Jiayin Pang, Tian-Peng Gao, Chun-Hui Zhang, Feng-Min Li, Neil C. Turner

**Affiliations:** 1 State Key Laboratory of Grassland Agro-ecosystems, School of Life Sciences, Lanzhou University, Lanzhou, 730000 Gansu Province China; 2 The UWA Institute of Agriculture and UWA School of Agriculture and Environment, The University of Western Australia, M082, Locked Bag 5005, Perth, WA 6001 Australia; 3 Centre of Urban Ecology and Environmental Biotechnology, Lanzhou City University, Lanzhou, 730070 China; 4 Northwest Plateau Institute of Biology, Chinese Academy of Sciences, Xining, 810008 Qinghai China

## Abstract

Seed germination behavior is an important factor in the distribution of species. Many studies have shown that germination is controlled by phylogenetic constraints, however, it is not clear whether phylogenetic constraints or environmental cues explain seed germination of a genus from a common ancestor. In this study, seed germination under different temperature- and water-regimes [induced by different osmotic potentials of polyethylene glycol (PEG)] was investigated in the phylogenetically-related *Caragana* species that thrive in arid, semiarid, semihumid and humid environments. The results showed that the final percentage germination (FPG) decreased from 95% in species from arid habitats to 0% in species from humid habitats, but with no significant phylogenetic signal. Rather, the response of seed germination to temperature and PEG varied greatly with species from arid to humid habitats and was tightly linked to the ecological niche of the species, their seed coat structure and abscisic acid concentration. The findings are not consistent with the hypothesis that within a family or a genus, seed germination strategies can be a stable evolutionary trait, thus constraining interspecific variation, but the results clearly show that seed germination of *Caragana* species distributed across a range of habitats has adapted to the environment of that habitat.

## Introduction

The ‘how and why’ of plant species’ distribution has been a subject of fundamental importance throughout the history of plant ecology^1^. Among several traits that account for species’ distribution, the germination of the seed is a critical step in the life cycle of a plant^2–4^ that is particularly vulnerable to environmental stress^3, 5, 6^. The most favorable period for seed germination of a species varies according to it’s geographic distribution and life cycle^7, 8^. Therefore, investigation of seed behaviour of species in response to a combination of biotic and abiotic factors may help to understand factors related to the distribution of the species^5^.

Among biotic factors, it is considered that seed germination is genetically determined and the phylogenetic signal is a significant constraint, termed a phylogenetic constraint^6, 9^, to the evolution and expression of seed traits. As a result of phylogenetic constraints, closely-related species do not move too far from their optimum niche and share similar seed-germination traits or niche preferences^6, 9, 10^. For example, when phylogenetic analysis is used to infer the evolution, there is strong evidence of phylogenetic constraints on seed size^11^. As seed size is positively correlated with mean time to germination, this suggests that seed germination is likely to be a phylogenetically-conserved trait^3^. Zhang *et al*.^6^ found that closely-related species shared similar germination times (temporal niche preferences). *Romulea* species that grow in different Mediterranean habitats exhibit strong phylogenetic constraints on the phenology of seed germination regardless of their habitat of origin^12^, and species in North American forests have germination strategies that match those of co-genetic species presently occurring in East Asia^13, 14^. These findings lend support to the hypothesis that within a family or a genus, germination strategies can be a stable evolutionary trait, thus constraining interspecific variation in germination^6, 9, 10, 12^. However, other studies have reported that seed germination differs within a family or a genus. For example, *Stellaria* species that grow in dry grasslands and in shady deciduous forests have some germinating seeds, while those that grow in open forest have completely dormant seeds at maturity in early summer^15^. Similar to *Stellaria* species, *Nothofagus* species along altitudinal gradient produce seeds that vary considerably in their germination^1^. These results, at least to some degree, indicate a smaller phylogenetic constraint on seed germination within a family or a genus, but how widespread this is still needs to be confirmed.

Among abiotic factors, temperature and water status are two of the most important factors that regulate seed germination^1, 16^. Studies have shown that the seed germination of species from distinct environments vary significantly in response to various temperature and water regimes^1, 8, 17–20^, that is, species have different optimal temperatures for seed germination and different abilities to germinate at low water potential^8, 16, 19^, the two main influences likely to account for species’ distribution^1^. For species from the same genus, seed germination traits adapt to local ecological factors, and species-specific germination preferences are different^15, 21, 22^. However, it is not clear whether seed germination, and consequently species’ distribution, of species that have evolved from the same ancestor, but diversified to distinct environments, have been strongly shaped by environmental cues.

The seed coat acts as a barrier to water permeability^23–25^, while abscisic acid (ABA) plays a important role in the induction and maintenance of seed dormancy^24, 26–28^ that can be reversed by gibberellic acid (GA_3_)^28^, so that both permeability and dormancy play an important role in seed germination. Therefore, if the phylogenetic signal constrains germination, seed coat structure and seed ABA content within a family or a genus are likely to be similar, while if environmental cues shape germination, they are likely to vary.

*Caragana* species have the same ancestor, but radiated from arid areas of northern China to humid and forested areas of East Asia and differentiated into different species depending on whether the habitats were arid, semiarid, semihumid or humid^29, 30^. These provide a valuable resource for a comparative study of the influence of phylogenetic constraints and environmental cues on seed germination. In this study, we hypothesized that over the wide range of environments, from arid to humid, in which the *Caragana* genus has evolved, the different species would exhibit great variation and small phylogenetic constraint in seed germination, thereby reflecting the distinct habitats and bioclimatic areas in which they occur. Specific questions were (1) whether phylogenetic constraints limited seed germination of *Caragana* species; if not, then: (2) whether the responses of seed germination to temperature and water status (using polyethylene glycol 6000) differed greatly, therefore reflecting the distinct habitats and bioclimatic environments in which they are distributed; if yes, (3) whether the variation in seed germination was related to differentiation in seed coat structure and ABA content; and (4) whether GA_3_ can reverse the effects of ABA.

## Results

### Site germination and phylogenetic signal

**Figure 1:**
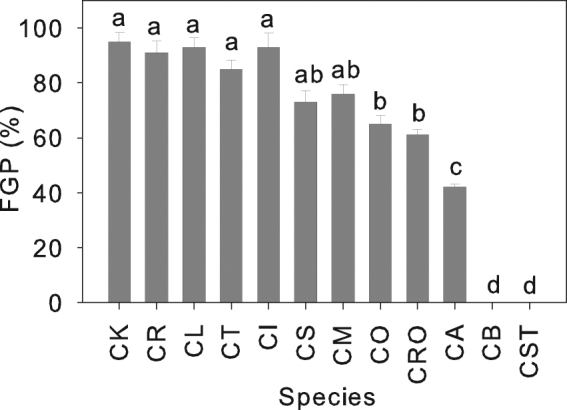
Final germination percentage (FGP) in seeds of 12 *Caranaga* species. Seeds of four species [*C*. *korshinskii* (CK), *C*. *roborovskyi* (CR), *C*. *leucophloea* (CL) and *C*. *tibetica* (CT)] from arid habitats, three species [*C*. *intermedia* (CI), *C*. *stenophylla* (CS) and *C*. *microphylla* (CM)] from semiarid habitats, four species [*C*. *opulens* (CO), *C*. *rosea* (CRO), *C*. *arborescens* (CA) and *C*. *boisi* (CB)] from semihumid habitats and one species [*C*. *stipitata* (CST)] from a humid habitat were incubated under well-watered conditions at the site of collection in Petri dishes covered with soil to keep the seeds in the dark. The mean daytime temperatures during germination varied from 17 to 20 °C. Data are means + one SE of the mean (*n* = 5). Species with different letters are significantly different from each other (*P* < 0.05).

Using the tetrazolium test, the ratio of viable seeds to total seeds in all *Caragana* species was greater than 90%. While the mean daytime temperature during germination varied from only 17 °C to 20 °C, the final germination percentage (FGP) of well-watered seeds at the sites of collection varied greatly among species, decreasing from 95% in species from arid habitats to 0% in species from humid habitats (Fig. 1). There was no significant phylogenetic signal in FPG (Table 1) based on observed Page’s λ = 0 (*P* = 0.99 against likelihood estimate with λ set to 0).

**Table 1:** Phylogenetic signal of seed germination traits based on the phylogenetic tree (Supplementary Fig. S1) of 12 *Caragana* species.

Trait	*n*	observed λ	*P*_0_ Value (against λ = 0)	*P*_1_ Value (against λ = 1)
FGP	12	0	0.99	<0.0001

*n*, number of species; observed λ, likelihood estimate of observed λ; *P*_0_, likelihood estimate with λ set to 0 (no phylogenetic signal); *P*_1_, likelihood estimate with λ set to 1 (maximum phylogenetic signal); FGP, final germination percentage. The phylogenetic signal was estimated as Pagel’s λ using a maximum likelihood framework (see Statistical analysis in Methods).

### Germination under controlled conditions

**Figure 2:**
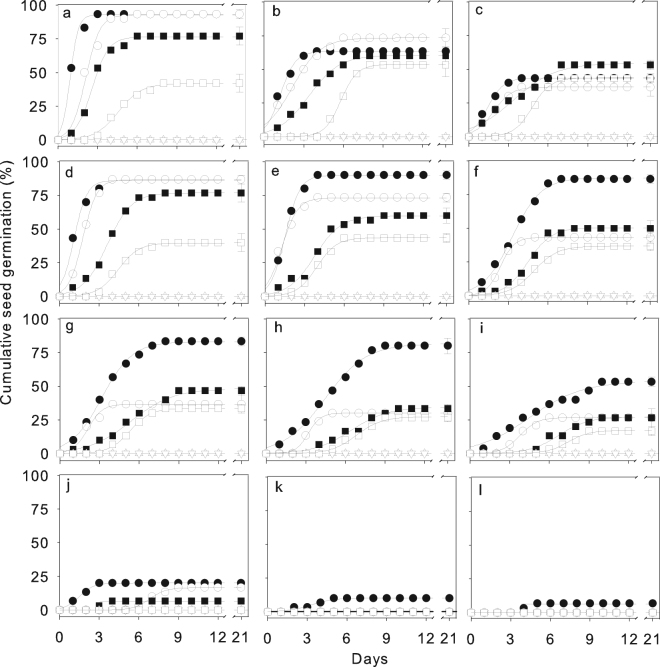
Cumulative seed germination of six C*aragana* species under different temperature- and water treatments. Seeds of species from arid (*C*. *korshinskii*, ●), semiarid (*C*. *intermedia*, ○, and *C*. *microphylla*, ■), semihumid (*C. arborescens*, □, and *C*. *boisi*, △), and humid (*C*. *stipitata*, ▽) habitats incubated at alternating (12/12 h) temperatures of 20/10 °C (**a**), 25/15 °C (**b**), 30/20 °C (**c**), and at water/osmotic potentials of PEG-6000 solution of 0.0 MPa (**d**), −0.2 MPa (**e**), −0.4 MPa (**f**), −0.6 MPa (**g**), −0.8 MPa (**h**), −1.0 MPa (i), −1.2 MPa (**j**), −1.6 MPa (**k**) and −1.8 MPa (**l**) at 20/10 °C. No seeds of *C*. *boisi* and *C*. *stipitata* absorbed water and germinated at any temperature and water/osmotic potential, and no seeds germinated in any species at a water/osmotic potential of −2.0 MPa (data not shown). Data are means ± one SE of the mean of the final germination percentage when larger than the symbol (*n* = 5); the lines are the fitted logistic curve (see Methods).

The FGP of the six *Caragana* species varied significantly with the temperature regime and the water/osmotic potential of the PEG solution and there was significant interaction between species and temperature regime and species and water/osmotic potential (Table 2). At 20/10 °C, FGP gradually decreased from 95% to 42%, and the time required for seeds to reach 50% of final FGP (T_50_) increased gradually from 0.9 d to 4.6 d in the species from an arid habitat, *C*. *korshinkii*, to the species from a semihumid habitat, *C*. *arborescens*. With increasing temperature, FGP decreased dramatically in species from arid and semiarid habitats, but not in *C*. *arborescens*, a species from a semihumid habitat, in which T_50_ also did not change significantly (Figs 2 and S2, Table S1). As the water/osmotic potential decreased, FGP of *C*. *korshinskii*, the species from an arid habitat, did not decrease significantly until −0.8 MPa, then decreased to 20% at −1.2 MPa and to 6% at −1.8 MPa, while FGP in the species from semiarid and semihumid habitats decreased significantly as the water/osmotic potential decreased below −0.2 MPa, so that no seeds germinated at −1.6 MPa in the species from a semiarid habitat and at −1.2 MPa in the species from a semihumid habitat (Figs 2 and S3). T_50_ tended to increase with the decreasing water/osmotic potential of the PEG solution (Table S1). Seeds of *C*. *boisi* and *C*. *stipitata*, did not absorb water and germinate at any temperature or PEG treatment (Figs 2, S2 and S3, Table S1).

**Table 2:** Analysis of the final germination percentage of the six *Caragana* species in relation to temperatures and their interaction, in relation to water/osmotic potential treatments and their interaction, and in relation to gibberellic acid treatments and their interaction.

Source of variance	df	MS	*F*-value	*P*-value
**Temperature**				
Species (S)	5	5462	71.6	<0.001
Temperature (T)	2	1820	23.9	<0.001
S × T	10	618	8.2	<0.001
**Water/osmotic potential**				
Species (S)	5	8982	232	<0.001
Water/osmotic potential (W)	9	9741	252	<0.001
S × W	45	526	13.6	<0.001
**Gibberellic acid (GA**_**3**_ **)**				
Species (S)	5	148.6	147.5	<0.001
GA_3_ concentration (C)	6	29.6	29.3	<0.001
S × C	30	4.6	4.5	<0.001

df, degrees of freedom; MS, mean squares; *F*-value, value of the F-statistic; *P*-value, probability.

**Figure 3:**
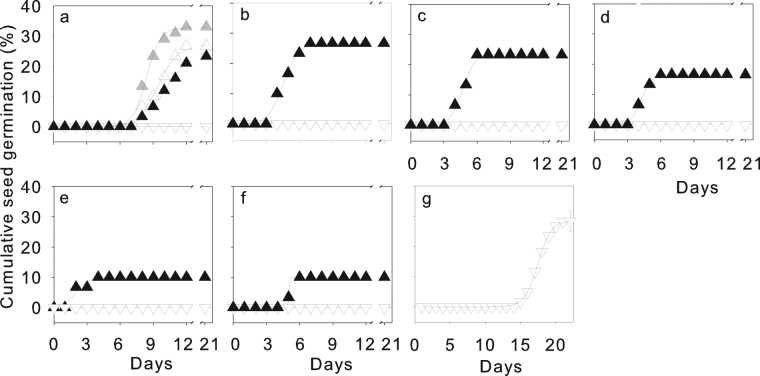
Cumulative germination of cracked seeds of *C*. *boisi* and *C*. *stipitata*. Cracked seeds of *C*. *boisi* (▲, △, ) and *C*. *stipitata* (▽) from a semihumid and humid habitat, respectively, incubated at alternating (12/12 h) temperatures of 20/10 °C (▲), 25/15 °C (△), 30/20 °C () at a water/osmotic potential of 0.0 MPa (**a**), and water/osmotic potentials of PEG-6000 solution of 0.0 MPa (**b**), −0.2 MPa (**c**), −0.4 MPa (**d**), −0.6 MPa (**e**), −0.8 MPa (**f**) at 20/10 °C. Cracked seeds of *C*. *stipitata* did not germinate at any temperature and water/osmotic potential, and no seeds germinated in *C*. *boisi* at water/osmotic potentials below −0.8 MPa (data not shown). Cumulative germination of cracked seeds of *C*. *stipitata* washed with water for 3 h each day during the incubation period (**g**). Data are means ± one SE of the mean of the final germination percentage when larger than the symbol (*n* = 5); the lines are the fitted logistic curve (see Methods).

Cracked seeds of *C*. *boisi* germinated, and FGP reached about 23% at 20/10 °C, increased slightly with warmer temperatures (Fig. 3a), and decreased dramatically with a decrease in the water/osmotic potential of the PEG solution (Fig. 3b–f, Table S2). However, cracked seeds of *C*. *stipatata* did not geminate and heat shock, cold stratification, smoking and ethanol treatment did not induce *C*. *stipatata* seeds to germinate, but the 3-h daily washing treatment induced about 30% of the seeds to germinate (Fig. 3g).

**Figure 4:**
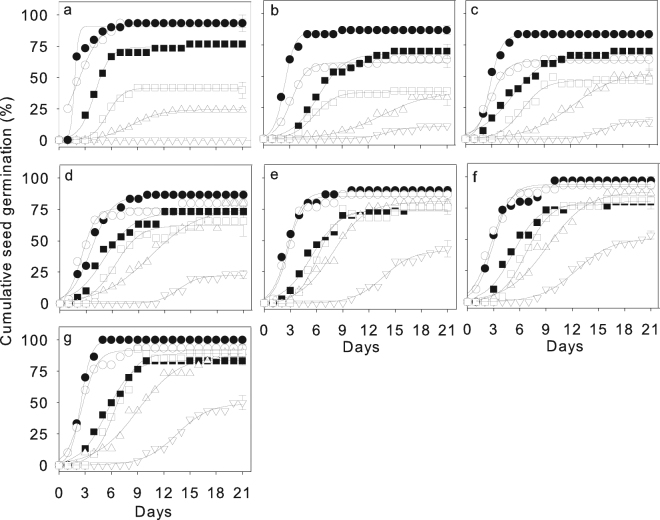
Cumulative seed germination of six *Caragana* species under gibberellic acid treatments. Seeds of species from arid (*C*. *korshinskii*, ●), semiarid (*C*. *intermedia*, ⚬, and *C*. *microphylla*, ■), semihumid (*Caragana arborescens*, □, and *C*. *boisi*, △), and humid (*C*. *stipitata*, ▽) habitats incubated with gibberellic acid (GA_3_) concentrations of 0 μg g^−1^ (**a**), 100 μg g^−1^ (**b**), 250 μg g^−1^ (**c**), 500 μg g^−1^ (**d**), 1000 μg g^−1^ (**e**), 1500 μg g^−1^ (**f**), 2500 μg g^−1^ (**g**) at 20/10 °C. The seed coat of *C*. *boisi* and *C*. *stipitata* was cracked. Data are means ± one SE of the mean of the final germination percentage when larger than the symbol (*n* = 5); the lines are the fitted logistic curve (see Methods).

The FGP of the six *Caragana species* was significantly affected by species, GA_3_ and their interaction (Table 2). The GA_3_ solution increased the FGP of all the species, especially *C*. *boisi* and *C*. *stipatata* at high GA_3_ concentrations (Fig. 4). Compared with the 0 μg g^−1^ control, T_50_ increased when the seeds were treated with 100 μg g^−1^, but tended to decrease with increasing GA_3_ concentration (Figs 4 and S4, Table S3) in all species.

### Seed coat structure and ABA content

**Figure 5:**
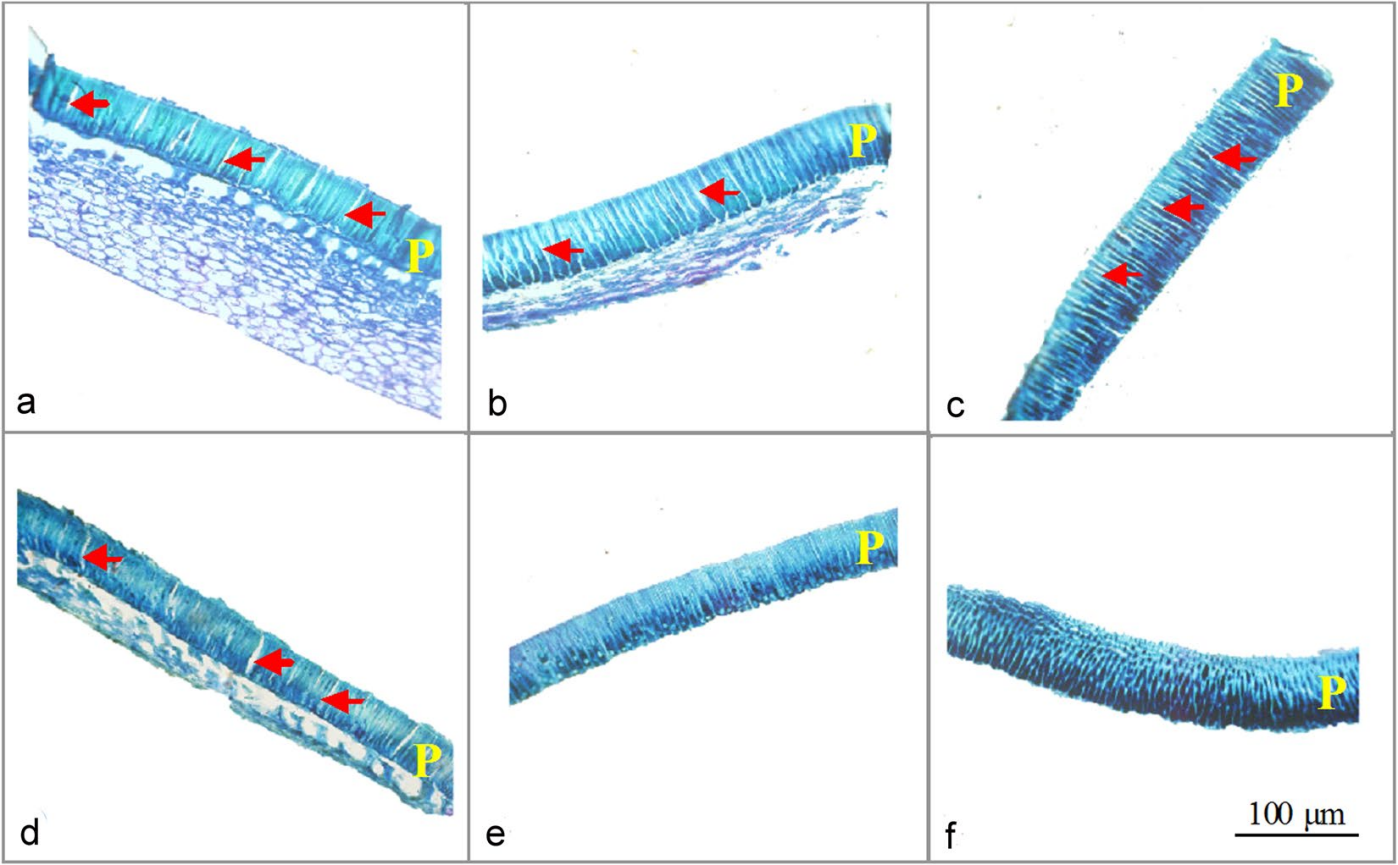
Seed coat structure. Micrographs using light microscopy of the seed coat structure of six *Caragana* species from arid (*C*. *korshinskii*, **a**), semiarid (*C*. *intermedia*, **b**, and *C*. *microphylla*, **c**), semihumid (*C*. *arborescens*, d, and *C*. *boisi*, **e**) and humid (*C*. *stipitata*, **f**) habitats. Arrows show the gaps in the palisade tissue in (**a**) to (**d**); P, palisade layer.

The seed coat consisted mainly of a palisade layer composed of macrosclereids, with gaps between the macrosclereids in *C*. *korshinskii*, *C*. *intermedia*, *C*. *microphylla* and *C*. *arborescens*, but with no gaps between the macrosclereids in *C*. *boisi* (Fig. 5). For *C*. *stipatata*, the palisade layer was composed of brachysclereids, with no gaps between the brachysclereids (Fig. 5).

**Figure 6:**
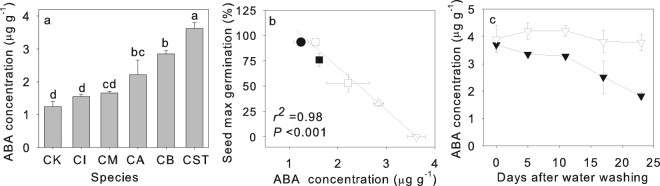
ABA concentration, the correlations between ABA concentration and seed maximum germination percentage of six *Caragana* species and ABA concentration after daily washing in *C*. *stipitata*. (**a**) ABA concentration of six *Caragana* species from arid (*C*. *korshinskii*, CK), semiarid (*C*. *intermedia*, CI, and *C*. *microphylla*, CM), semihumid (*C*. *arborescens*, CA, and *C*. *boisi*, CB) and humid (*C*. *stipitata*, CST) habitats, (**b**) relationship between ABA concentration and maximum final seed germination of each species (*C*. *korshinskii*, ●, *C*. *intermedia*, ○, *C*. *microphylla*, ■, *C*. *arborescens*, □, and cracked seeds of *C*. *boisi*, △ and *C*. *stipitata*, ▽), and (**c**) change with time in the ABA concentration of cracked seed after daily washing with (▼) and without (▽) water in *C*. *stipitata*. Data are means + one SE (**a**) or ± one SE (**b**,**c**) of the mean of the final germination percentage when larger than the symbol (*n* = 5). In (**b**), the line is the fitted linear regression with the correlation coefficient (*r* ^2^) and probability values (*P*) given.

The ABA concentration in the seeds ranged from 1.2–1.6 μg g^−1^ in *C*. *korshinskii*, *C*. *intermedia* and *C*. *microphylla*, 2.2–2.8 μg g^−1^ in *C*. *arborescens* and *C*. *boisi*, and 3.7 μg g^−1^ in *C*. *stipatata* (Fig. 6a). Across the six species, there was a significant negative relationship between maximum FGP and ABA concentration (Fig. 6b). The ABA concentration in cracked seeds of *C*. s*tipatata* decreased gradually with daily washing with water (Fig. 6c) and as the seeds germinated (Fig. 3g).

## Discussion

The uplift of the Qinghai-Tibetan Plateau, especially the onset of the Himalayan uplift, is considered to have induced the formation of an arid climate in northwest China^31^, and the latter, in turn, induced the origin of *Caragana* in the steppe areas. Subsequently the ancestor of *Caragana* radiated from the arid areas of northwest China to the humid and forested areas of East Asia^29, 30^, where they differentiated into species with different ecological niches^30, 32^. The general consensus from a number of studies is that a phylogenetic signal in both functional traits and niche preference is fairly common in plants^3, 9, 33–35^, and seed germination is largely influenced by such a phylogenetic signal. That is, germination strategies can be stable evolutionary traits that do not vary with environment and remain similar within a genus or family^3, 6, 10–14^. However, other studies have reported that species within a family or a genus develop different seed germination behaviors that depend on the local environment^1, 15^. In this study, we used a comparative analysis of the germination behavior of the *Caragana* species in their natural habitat as a test for phylogenetic constraints. The results showed that FGP decreased from more than 95% in the species from an arid habitats to 0% in the species from a humid habitat and, based on Pagel’s λ, seed germination does not represent a strongly-conserved phylogenetic niche in the genus, but has adapted to the environments in which the different species evolved.

In arid and semiarid regions, it is presumed that seed germination in summer bears a high risk of seedling loss due to long periods of drought and high temperature, and that most species in these regions appear to avoid germination in summer and autumn^17^. However, 70–95% of the mature seeds in *Caragana* species from arid and semiarid areas germinated within 2–4 d after the beginning of incubation (high FGP, low T_50_). We suggest that the reason for the rapid germination is that the regions where *Caragana* species occur are influenced by the east-Asian monsoon, which results in more than 70% of the annual precipitation falling during the May–September growing season^31^, while only 40–60 mm of the annual precipitation falls in winter and spring. To ensure population regeneration, the timing of seed germination needs to coincide with a period of rainfall and the seedlings need to have a reasonable growth period after germination to grow large and acquire resources for subsequent survival and growth^5^. Therefore, *Caragana* species from arid and semiarid habitats are early flowering, have early seed maturation, and early and rapid seed germination after maturity in summer. The present results suggest these species use ‘opportunistic’ strategies to ensure rapid emergence and establishment after only one rainfall event^36^. In addition, high temperatures in summer in these areas result in high rates of transpiration and drought stress. Therefore, seed germination has adapted to rainfall-induced low temperature (increasing FGP in response to low temperature), and the ability to tolerate drought stress (germination even at osmotic potentials of −1.2 MPa). Further, as almost all seeds are eaten by seed predators within one week of falling to the ground, there is no advantage for seeds of plants from arid and semiarid habitats delaying germination until the beginning of in the next growing season^37^.

In semihumid and humid environments the requirements for seed germination are different. *C*. *arborescens*, a species from a semihumid habitat, had a permeable seed coat and absorbed water, but the FGP was about 50%, while seeds of *C*. *boisis*, the other species from a semihumid habitat, and seeds of *C*. *stipitata*, a species from a humid habitat, had an impermeable seed coat and did not germinate. The results from this study suggest that germination decreased (increasing degree of dormancy) with increasing precipitation^36^. *C*. *arborescens* is found at altitude ~200 m, where the air temperature is relatively high when the seeds mature, and the length of the growing season for seedlings is relatively long (from early August to mid-October). Therefore, some mature seeds germinate when the conditions are suitable. On the other hand, *C*. *boisi* is found on the south-eastern Tibetan Plateau, and *C*. *stipitata* grows at the top of the Hua mountains, where the altitude is more than 1800 m. The time for peak flowering of the two species is in early June and seeds mature in mid-August, more than two months later than those of the species in arid habitats. Thus, if germination were to occur, the period for seedling growth would be very short and low temperatures and/or early frost would kill the seedlings^14, 17, 38, 39^. Therefore, the seeds of *C*. *boisi* and *C*. *stipitata* have strong seed dormancy when mature, but germination in the following growing season would be advantageous as water is not a limiting factor (700–1000 mm precipitation per year, 70% in the growing season). Accordingly, seed germination was sensitive to drought stress (decreasing water/osmotic potential) and tended to increase with increasing temperature. Besides temperature and water status, other factors, such as light, may influence seed germination, and the investigation of their influence on seed germination would be beneficial.

The seed coat acts as a barrier to water permeability as a result of the arrangement (how tightly packed) and chemical coating/impregnation of cells in the palisade layer^23–25^. For seeds to germinate, the water-impermeable layers of the seed coat must become permeable to allow passage of water to the embryo. In this study, we found there are a number of gaps between the macrosclereids in the seed coat of species from arid, semiarid and one semihumid habitat, enabling the seeds to absorb water and germinate, while there were no gaps between the macrosclereids/brachysclereids in the seed coat of the species from the other semihumid habitat and the humid habitat, the seeds did not absorb water and were dormant. Therefore, the findings showed that divergence in seed coat structure is important in determining whether the seed germination behavior in *Caragana* species is ‘opportunistic’ or dormancy-induced. To enable germination of seeds without gaps in the palisade layer, we suggest that after dispersal they form a seed bank in the soil and mechanical damage and/or abrasion of the seed coat by soil disturbance and microbial action result in the development of cracks in the seed coat that results in germination^24, 40, 41^.

Many studies on a wide variety of plant species have demonstrated abscisic acid (ABA) is a positive regulator of the induction of dormancy during seed maturation, and a negative regulator of seed germination^4, 28^ as ABA suppresses differential and mitotic cell division^27^. For example, *Arabidopsis* mutants that over-accumulate ABA have enhanced dormancy levels or delays in germination^42^, but mutants that lack the capacity to produce ABA showed the absence of primary dormancy in mature seeds, while some ABA-insensitive mutants also lack, or have decreased, primary dormancy in mature seeds^43^. Further, in tomato, higher ABA levels enhanced dormancy levels, and ABA deficiency resulted in non-dormancy^44^. A direct correlation between dormancy and high endogenous ABA content has also been reported in tree seeds^45^. In this study, the maximum FGP (after eliminating the seed coat effect) decreased linearly with an increase in seed ABA concentration, suggesting that ABA concentration might determine physiological dormancy in *Caragana* species, and consequently result in different FGP in different species. In order to test this, a range of GA_3_ treatments was applied, as gibberellins (GA) have the opposite effect to ABA and promote seed germination^28, 46^. The results showed that GA_3_ promoted seed germination, especially in species from semihumid and humid habitats, supporting the conclusion that ABA is regulating seed dormancy and seed germination in *Caragana*. The findings are consistent with previous studies showing that exogenous application of gibberellic acid (GA_3_) to intact, unstratified seeds is effective in breaking the dormancy of seeds of *Myrica esculenta* and *Phellodendron amurense* ^45, 47^. Furthermore, the findings also showed that, besides seed coat structure, seed ABA concentration is a key factor determining dormancy-induced seed germination in *Caragana* species. Seeds of *C*. *stipitata* have strong dormancy and usually form a seed bank in the sand and shallow soil on the top of the Hua mountains. Due to frequent rainfall, they are regularly washed by water, which may be an effective means of breaking dormancy, as cracked seed in combination with washing with water decreased the ABA concentration in the seed and increased germination.

In conclusion, the results presented in this study demonstrate that there was no significant phylogenetic signal in the germination of *Caragana* species. Seeds of species from arid and semiarid habitats lacked dormancy, seeds of species from semihumid habitats had physiological dormancy or physical (seed coat) and physiological dormancy, while seeds of species from humid habitats had physical and physiological dormancy. Furthermore, variation in seed germination of *Caragana* species was related to seed coat structure and ABA concentration. The results provide a better understanding of the existing patterns of species’ distribution and adaptation. However, as global climate change is altering the environmental conditions^48^, the strong environmental cues in seed germination behavior shown in this study suggest that climate change will undoubtedly affect seed germination, and subsequently recruitment of plants and population dynamics.

## Methods

### Plant materials

*Caragana* species are all early-flowering, small trees or woody perennial shrubs. They occur in distinct habitats from desert to deciduous woodland environments along an annual precipitation gradient from 100 mm to 1000 mm^29, 30, 49–52^ (Table S4, Fig. S5, for further details see Supplementary Information Appendix S1). The period of seed maturation gradually becomes later in more humid environments, from mid-June in species from arid habitats to mid-August in the species from humid habitats (Fig. S6). The seed mass of the species was similar (between 12 and 38 mg seed^−1^) with the exception of *C*. *korshinskii* which has a larger seed of over 80 mg seed^−1^ (Table S4).

From June to August 2012, seeds of the 12 *Caragana* species were collected from more than 20 unique individual plants each at physiological maturity when the pods change colour from green to brown. Ripe pods were spread out to allow them to open and the seed removed. Some seeds were germinated at the site where they were collected under natural conditions and the remaining seeds were transported to the laboratory where they were used to assess their viability following the tetrazolium test procedure described by Santos *et al*.^53^, and germinated under control conditions.

### Experiment 1- natural site germination and phytogenetic tree

Seeds of the 12 species were germinated under the natural environmental temperatures at the site of collection in three-fold filter paper in 90-mm diameter by 15-mm deep Petri dishes covered with soil to keep the seeds in dark. Distilled water was added to each Petri dish until the seeds floated, but were not inundated, and fresh distilled water was added each day. Each species had five replications of 30 seeds. The experiment continued until FGP became constant at which time the FGP was recorded. The mean daytime temperature during the seed incubation period was recorded at a weather station near to each site.

A phylogenetic tree of the 12 species was constructed based on three commonly sequenced chloroplast gene regions: ITS, rbcL and trnS-trnG DNA sequences were retrieved from the GenBank of NCBI for 11 of the *Caragana* species, or were isolated from fresh leaves for *C*. *intermedia*; *Hedysarum alpinum*, a member of a sister species to *Caragana* was used to establish the relationship between the *Caragana* species (Table S5). The three markers were amplified by polymerase chain reaction (PCR) with primers published in literature^29^. The sequences of the three genes were aligned using ClustalW (http://www.ebi.ac.uk/clustalw/), followed by manual adjustments in BioEdit v7.0.9 (Carlsbad, CA). The phylogenetic tree was built through the MEGA software package (version 5.0, www.megasoftware.net).

### Experiment 2- Seed germination under controlled conditions

From the 12 species used in Experiment 1, one typical wide-spread species from an arid habitat, *C*. *korshinskii*, two typical wide-spread species from semiarid habitats, *C*. *intermedia* and *C*. *microphylla*, two species from semihumid habitats, *C*. *arborescens* and *C*. *boisi*, and one species from a humid habitat, *C*. *stipitata*, were chosen for seed germination under controlled conditions in the laboratory. To minimize the effect of after-ripening, the experiments were started shortly after the seeds had been transported to the laboratory from their sites of collection. Intact plump seeds were surface sterilized for 600 s with 75% ethanol. They were then placed on three-fold filter paper in 90-mm diameter by 15-mm deep Petri dishes and distilled water, or a solution of polyethylene glycol (PEG) 6000, or gibberellic acid (GA_3_), was added to each dish until the seeds floated, but were not inundated. Each treatment had five replications of 30 seeds.

To examine the effects of temperature on seed germination, the seeds were incubated in distilled water, which was added as required to maintain the depth as on the first day, in separate incubators with alternating (12/12h) temperature regimes of 20/10 °C, 25/15 °C, 30/20 °C and kept in constant darkness. The number of germinating seeds was recorded daily and the experiment continued until FGP became constant.

To examine the effects of water status on seed germination, seeds were incubated with solutions of PEG-6000 of known osmotic potential, 0, −0.2, −0.4, −0.6, −0.8, −1.0, −1.2, −1.6, −1.8, −2.0 MPa at 20 °C^54^, completely randomized within each incubator, and maintained at an alternating (12/12 h) temperature of 20/10 °C. Each day the solution in each Petri dish was removed, a further 10 ml test solution added and removed again as completely as possible, before the test solution was added as required to maintain the depth as on the first day^55^. The experiment continued until FGP became constant.

As seeds of *C*. *boisi* and *C*. *stipatata* did not absorb water and failed to germinate in the above treatments, the structure of the seed coat of the six species was analyzed by m**i**croscopic observation to compare differences between species in which the seeds absorbed water with species in which the seeds did not absorb water. Then, in order to eliminate the effect of seed coat impermeability on FGP, the seed coat of *C*. *boisi* and *C*. *stipatata* was cracked (cracked seeds) before germination under the three temperature and ten water status regimes above.

Cracked seeds of *C*. *boisi* germinated, but cracked seeds of *C*. *stipatata* still failed to germinate. To stimulate the break of dormancy of the *C*. *stipatata* seeds, a heat shock treatment, a cold stratification treatment, a smoke treatment and an ethanol treatment were applied (details in Supplementary Information Appendix S2). In addition, cracked seeds of *C*. *stipatata* were either washed for 3 h each day in running water at 20 °C (washing treatment) or not washed during the incubation period. After each of these treatments, seeds were germinated in an incubator at 20/10 °C as described above. Cracked seeds (about 0.5 to 1.0 g dry weight) with and without washing for 3 h each day were randomly removed at 0, 5, 11, 17, and 21 days during incubation to measure the abscisic acid (ABA) concentration of the seeds.

To examine the effect of GA_3_ on seed germination, seeds of *C*. *korshinkii*, *C*. *intermedia*, *C*. *microphylla* and *C*. *arborescens*, and cracked seeds of *C*. *boisi* and *C*. *stipitata* were incubated with solutions of GA_3_ of 0, 100, 250, 500, 1000, 1500, 2000, 2500 μg g^−1^ at an alternating (12/12 h) temperature of 20/10 °C. The solution was added in the same way as the PEG solutions. The number of germinating seeds was recorded daily and the experiment continued until FGP became constant.

The cumulative seed germination data were fitted by a logistic curve^56^:

$${\rm{GP}}={\rm{A}}/[1+\exp (-({\rm{t}}-{{\rm{t}}}_{0})/{\rm{b}}]$$

in which GP is the germination percentage at *t* time (days), *A* estimates the FGP, t_0_ is the time required for each replicate set of seeds to reach 50% of final FGP and b is related to the rate of seed germination.

### Microscopic observations

To determine the structure of the seed coat, seeds were embedded in epoxy resin after fixing in 4% FAA solution (formaldehyde, 70% ethanol and acetic acid), and transverse and longitudinal sections approximately 8–10 µm thick were cut with a microtome. These sections, stained by an aqueous solution of 1% toluidine blue, were observed using a light microscope (Axioskop II plus; Zeiss, Oberkochen, Germany) and photographed with a digital camera (Nikon Digital Sight, Nikon, Tokyo, Japan).

### ABA determination

Seed ABA was extracted following the methods described by Tombesi *et al*.^57^. Analyses were performed on an Agilent 1260 HPLC (Agilent Ltd., California, USA) equipped with an Agilent C18 ZORBAX (5 mm × 150 mm × 4.6 mm) column at a flow rate of 0.013 ml s^−1^. The injection volume was 10 μL and the detection was made at 265 nm. The mobile phase of acetonitrile/methanol/water (8:40:52 v/v/v, 0.6% acetic acid) was previously filtered and degassed. ABA was identified by comparing the retention times with those of standard ABA, and the peak area quantified by an external standard method. Stock solutions of ABA standards was prepared by diluting a solution (0.1 mg mL^−1^ in acetonitrile) to obtain a range of concentrations from 0.1 to 10 μg mL^−1^.

### Statistical analysis

The phylogenetic signal in FGP of species was tested by estimating Pagel’s λ with the ‘fitContinuous’ function in the R package ‘geiger’ v1.99-3^58^, which uses a maximum likelihood framework to estimate the parameter λ. Pagel’s λ measures correlations relative to the correlation expected under Brownian movement^59^ and can vary from 0 (no phylogenetic signal) to 1 (strong phylogenetic signal)^6^. We tested for the significance of phylogenetic signal against the assumption of no signal (λ = 0) and strong signal (λ = 1) using a likelihood-ratio test^58^.

The germination data were analyzed by analysis of variance (ANOVA) using SPSS 15.0 (SPSS, Chicago, IL). Data were log-transformed to meet the requirement of normal distribution. Mean values for treatments were compared using least significant differences (LSD) at *P* = 0.05. A logistic curve was fitted for the relationship between cumulative FGP with germination days using Sigmaplot 10.0 (Systat Software, Inc., Chicago, IL, USA). Maximum final seed germination of six species (using cracked seeds in *C*. *boisi* and *C*. *stipitata*) among temperature- and water-regime treatments were used to fit linear regressions with seed ABA content.

### Data availability statement

All data generated or analysed during this study are included in this published article (and its Supplementary Information files).

## Electronic supplementary material

Supplemental Materials
