# Supplementary material for: Seed germination of Caragana species from different regions is strongly driven by environmental cues and not phylogenetic signals
Source: Sci Rep. 2017 Sep 12;7:11248. doi: 10.1038/s41598-017-11294-x (PMC5596004; doi:10.1038/s41598-017-11294-x)
Supplement: Supplementary file 1 — Supplemental Materials [file 41598_2017_11294_MOESM1_ESM.doc]

**Supplemental Materials**

**Seed germination of *Caragana* species from different regions is strongly driven by environment cues and not phylogenetic signals**

Xiang-Wen Fang, Juan-Juan Zhang, Dang-Hui Xu, Jiayin Pang, Tian-Peng Gao, Chun-Hui Zhang, Feng-Min Li and Neil C. Turner

**Appendix S1.** *Caragana* **s**pecies selection and habitat determination:

*Caragana* species occur in distinct habitats from desert to deciduous woodland environments along an annual precipitation gradient from 100 mm to 1000 mm. *C. korshinskii, C. roborovskyi*, *C. leucophloea* and *C. tibet* are mainly restricted to arid regions in western Inner Mongolia and/or in the middle of the Hexi Corridor in Gansu Province where the annual precipitation is less than 200 mm. *C. intermedia*, *C. stenophylla* and C. *microphylla* are mainly restricted to semiarid regions of middle and eastern China where the annual precipitation is between 200 mm and 400 mm, *C. opulens*, *C. rosea*, *C. boisi* and *C. arborescens* are mainly restricted to the semihumid region of central and eastern China where the annual precipitation is between 400 mm and 800 mm and *C. stipitata* is mainly restricted to the humid Hua mountains where the annual precipitation is 1000 mm^1-8^. Due to aerial seeding and manual planting for revegetation purposes, *C. korshinskii* is also found in semiarid regions^1^. While some plants of *C. roborovskyi* and *C. rosea* occur in semiarid regions, some plants of *C. stenophylla* occur in arid regions, and some plants of *C. microphylla* can be found in semihumid regions, the majority of plants of *C. korshinskii* and *C. roborovskyi* are distributed in arid regions^2^, *C. stenophylla* and *C. microphylla* in semiarid regions^3,4,5,6,7^ and *C. rosea* in semihumid regions^8^. Therefore their habitats were determined according to the region in which most plants are naturally distributed.

**Appendix S2.** Physico-chemical treatments to break dormancy in *C. stipitata* seeds:

Seeds of *C. stipatata* failed to germinate even after the seed coat was cut (cracked seed). To stimulate the break of dormancy, physico-chemical treatments were applied as followings: (1) seeds were heated in a forced convection oven at 50 °C, 70 °C, 90 °C, 110 °C, 130 °C for 300 s (heat shock treatment). This temperature range has been shown to be effective in breaking dormancy for a great many plant species^9^; (2) seeds were maintained at 4 °C in a refrigerator for 6, 12 and 18 months in wet sand, and then the coat of seeds was cracked (cold stratification treatment); (3) seeds were immersed for 3 h in an aqueous solution of smoke following the method described by Keeley and Fotheringham^10^ (smoke treatment); or (4) cracked seeds were incubated in ethanol (4% v/v) rather than distilled water (ethanol treatment). After treatment, the seeds were incubated at 20/10 °C to see whether the treatments stimulated seed germination.

**References**

1. Fang, X. W. *et al*. Limits to the height growth of *Caragana korshinskii* resprouts. *Tree Physiol.* **33**, 275–284 (2013).

2. Xie, L. N., Ma, C. C., Guo, H. Y., Li, Q. F. & Gao, Y. B. Distribution pattern of *Caragana* species under the influence of climate gradient in the Inner Mongolia region, China. *J. Arid Land* **6**, 311–323 (2014).

3. Xie, LN. Gou HY & Ma CC. Alterations in flowering strategies and sexual allocation of *Caragana stenophylla* along a climatic aridity gradient. *Sci. Rep.* **6**, 33602; doi: 10.1038/srep33602 (2016).

4. Ma, C. C., Gao, Y. B., Guo, H. Y. & Wang, J. L. Interspecific transition among *Caragana microphylla, C. davazamcii* and *C. korshinskii* along geographic gradient. I. Characteristics of photosynthesis and water metabolism. *Acta Bot. Sin.* **45,** 1228–1237 (2003).

5. Ma, C. C., Gao, Y. B., Guo, H. Y. & Wang, J. L. Photosynthesis, transpiration, and water use efficiency of *Caragana microphylla*, *C. intermedia*, and *C. korshinskii*. *Photosynthetica* **42**, 65–70 (2004).

6. Ma, C. C. *et al*. Physiological adaptations of four dominant *Caragana* species in the desert region of the Inner Mongolia Plateau. *J. Arid Environ.* **72**, 247–254 (2008).

7. Fang, X. W. *et al*. The distribution of four *Caragana* species is related to their differential responses to drought stress. *Plant Ecol.* **215**, 133–142 (2014).

8. Chang, Z. A taxonomical study of *Caragana* Fabr. From China. Dissertation for the Degree of Doctor. Northeast Forestry University (2008).

9. Fotheringham, K. Trace gas emissions and smoke-induced seed germination. *Science* **276**, 1248-1250 (1997).

10. Keeley, J.E. & Fotheringham, C. Smoke-induced seed germination in California chaparral. *Ecol.* **79**, 2320–2336 (1998).

Table S1. The time required for seeds to reach 50% of FGP (T_50_) in six *Caragana* species in three different alternating (12/12 h) temperature regimes and nine different water/osmotic potential solutions of PEG 6000. Values were obtained from the fitted values of the data in Fig. 2.

| Species | T_50_ (d) | | | | | | | | | | | |
| --- | --- | --- | --- | --- | --- | --- | --- | --- | --- | --- | --- | --- |
|  | 20/10 °C | 25/15 °C | 30/20 °C | 0  MPa | -0.2 MPa | -0.4 MPa | -0.6 MPa | -0.8 MPa | -1.0 MPa | -1.2 MPa | -1.6 MPa | -1.8 MPa |
| *C. korshinskii* | 0.9^cC^ | 1.2^bD^ | 1.4^aC^ | 1.9^cB^ | 1.5^cB^ | 3.3^bB^ | 3.2^bB^ | 4.5^aB^ | 4.5^aB^ | 1.5^cB^ | 3.3^b^ | 3.2^b^ |
| *C. intermedia* | 2.1^aB^ | 2.1^aC^ | 1.8^bC^ | 1.8^dB^ | 1.3^dB^ | 2.4^cC^ | 2.1^cC^ | 3.2^bC^ | 3.8^bB^ | 6.8^aA^ | - | - |
| *C. microphylla* | 2.6^bB^ | 3.2^aB^ | 3.4^aB^ | 3.6^cA^ | 3.7^cA^ | 4.5^bA^ | 5.3^bA^ | 6.4^aA^ | 6.7^aA^ | 2.4^dB^ | - | - |
| *C. arborescens* | 4.6^bA^ | 5.8^aA^ | 4.8^bA^ | 4.4^cA^ | 3.9^cA^ | 4.9^bA^ | 5.3^bA^ | 6.8^aA^ | 7.4^aA^ | - | - | - |
| *C. boisi* | - | - | - | - | - | - | - | - | - | - | - | - |
| *C. stipitata* | - | - | - | - | - | - | - | - | - | - | - | - |

Means within the same row in three different alternating (12/12 h) temperature regimes and nine different water/osmotic potential solutions of PEG 6000 followed by different lower-case letters or within the same column followed by different upper-case letters are significantly different at *P* < 0.05.

Table S2. The time required for seeds to reach 50% of final FGP (T_50_) in cracked seeds of *C. biosi* and *C. stipitata* in three different alternating (12/12 h) temperature regimes and nine different water/osmotic potential solutions of PEG 6000. Values were obtained from the fitted values of the data in Fig. 3.

| Species | T_50_ (d) | | | | | | | | | | | |
| --- | --- | --- | --- | --- | --- | --- | --- | --- | --- | --- | --- | --- |
|  | 20/10 °C | 25/15 °C | 30/20 °C | 0  MPa | -0.2 MPa | -0.4 MPa | -0.6 MPa | -0.8 MPa | -1.0 MPa | -1.2 MPa | -1.6 MPa | -1.8 MPa |
| *C. boisi* | 10^a^ | 9.4^b^ | 8.4^c^ | 4.6^a^ | 4.6^a^ | 4.3^a^ | 2^b^ | 5^a^ | - | - | - | - |
| *C. stipitata* | - | - | - | - | - | - | - | - | - | - | - | - |

Means within the same row in three different alternating (12/12 h) temperature regimes and nine different water/osmotic potential solutions of PEG 6000 followed by different lower-case letters are significantly different at *P* < 0.05.

Table S3. The time required for seeds to reach 50% of final FGP (T_50_) in six *Caragana* species in seven different gibberellic acid (GA_3_) concentrations at 20/10 °C. Values were obtained from the fitted values to the data in Fig. 4.

| Species | T_50_ (d) | | | | | | |
| --- | --- | --- | --- | --- | --- | --- | --- |
|  | 0 μg g^-1^ | 100 μg g^-1^ | 250 μg g^-1^ | 500 μg g^-1^ | 1000 μg g^-1^ | 1500 μg g^-1^ | 2500 μg g^-1^ |
| *C. korshinskii* | 1.8^cC^ | 2.4^bC^ | 2.8^bC^ | 3.5^aD^ | 2.6^bC^ | 3^bD^ | 2.5^bD^ |
| *C. intermedia* | 1.3^bC^ | 3.0^aC^ | 2.8^aC^ | 2.5^aD^ | 2.7^aC^ | 2.7^aD^ | 2.6^aD^ |
| *C. microphylla* | 4.0^cB^ | 6.2^aB^ | 5.3^bB^ | 5.1^bC^ | 5.4^bB^ | 5.2^bC^ | 5.4^bC^ |
| *C. arborescens* | 5.6^cB^ | 5.8^cB^ | 6.5^aB^ | 6.4^aC^ | 6.1^bB^ | 6.7^aC^ | 6.4^aC^ |
| *C. boisi* | 8.4^cA^ | 13.6^aA^ | 12.3^aA^ | 10.4^bB^ | 7.8^cB^ | 9.1^bB^ | 9.1^bB^ |
| *C. stipitata* | - | 14.1^bA^ | 15.6^aA^ | 13.5^bA^ | 13.6^bA^ | 12.4^cA^ | 12^cA^ |

Means within the same row followed by different lower-case letters or within the same column followed by different upper-case letters are significantly different at *P* < 0.05.

Table S4. Mean annual precipitation (MAP), altitude, average seed mass (*n* = 30), seed maturation period and habitats of the twelve *Caragana* species studied. Annual precipitation is averaged from 1971 to 2000 (data after 2000 not supplied) from the China Meteorological Data Sharing Service system.

| Species | MAP  (mm) | Altitude (m) | Seed mass (mg) | Maturation period | Habitats |
| --- | --- | --- | --- | --- | --- |
|
| *C. korshinskii* | 110 | 1400 | 80.6±2.9 | Mid-June | Desert and semi-desert |
| *C. roborovskyi* | 110 | 1400 | 21.0±1.4 | Mid-June | Desert and semi-desert |
| *C. leucophloea* | 110 | 1260 | 14.7±0.3 | Mid-June | Gobi desert |
| *C. tibetica* | 110 | 1400 | 11.8±1.3 | End-June | Desert and semi-desert |
| *C. intermedia* | 250 | 1370 | 35.6±0.5 | End-June | Desert steppe |
| *C. stenophylla* | 290 | 990 | 12.9±0.8 | Mid-July | Steppe |
| *C. microphylla* | 340 | 990 | 38.1±0.2 | Mid-July | Steppe |
| *C. opulens* | 500 | 1900 | 15.9±0.8 | End-June | Shrubland |
| *C. rosea* | 510 | 1100 | 18.8±0.2 | Mid-June | Shrubland |
| *C. aborescens* | 720 | 200 | 29.4±0.2 | Early August | Woodland margins |
| *C. boisi* | 730 | 1800 | 21.9±0.4 | Mid-August | Deciduous woodland |
| *C. stipitata* | 1000 | 1810 | 25.0±0.2 | Mid-August | Deciduous woodland |

Table S5. Species list with accession numbers of DNA sequences from the GenBank of the 11 *Caragana* species and *Hedysarum alpinum*, a member of the sister group to the *Caragana* species.

| Species | *Its* | *rbcL* | trnS-trnG |
| --- | --- | --- | --- |
| *C. korshinskii* | FJ537266 | FJ537215 | FJ537168 |
| *C. roborovskyi* | FJ537254 | FJ537204 | FJ537156 |
| *C. leucophloea* | FJ537275 | FJ537224 | FJ537177 |
| *C. tibetica* | FJ537244 | FJ537195 | FJ537145 |
| *C. intermedia* | this study | this study | this study |
| *C. stenophylla* | FJ537274 | FJ537223 | FJ537176 |
| *C. microphylla* | FJ537264 | FJ537213 | FJ537166 |
| *C. opulens* | FJ537282 | FJ537231 | FJ537183 |
| *C. rosea* | FJ537272 | FJ537221 | FJ537174 |
| *C. arborescens* | FJ537262 | FJ537211 | FJ537164 |
| *C. boisi* | FJ537259 | FJ537208 | FJ537161 |
| *C. stipitata* | FJ537260 | FJ537209 | FJ537162 |
| *Hedysarum alpinum* | FJ537289 | FJ537237 | FJ537190 |


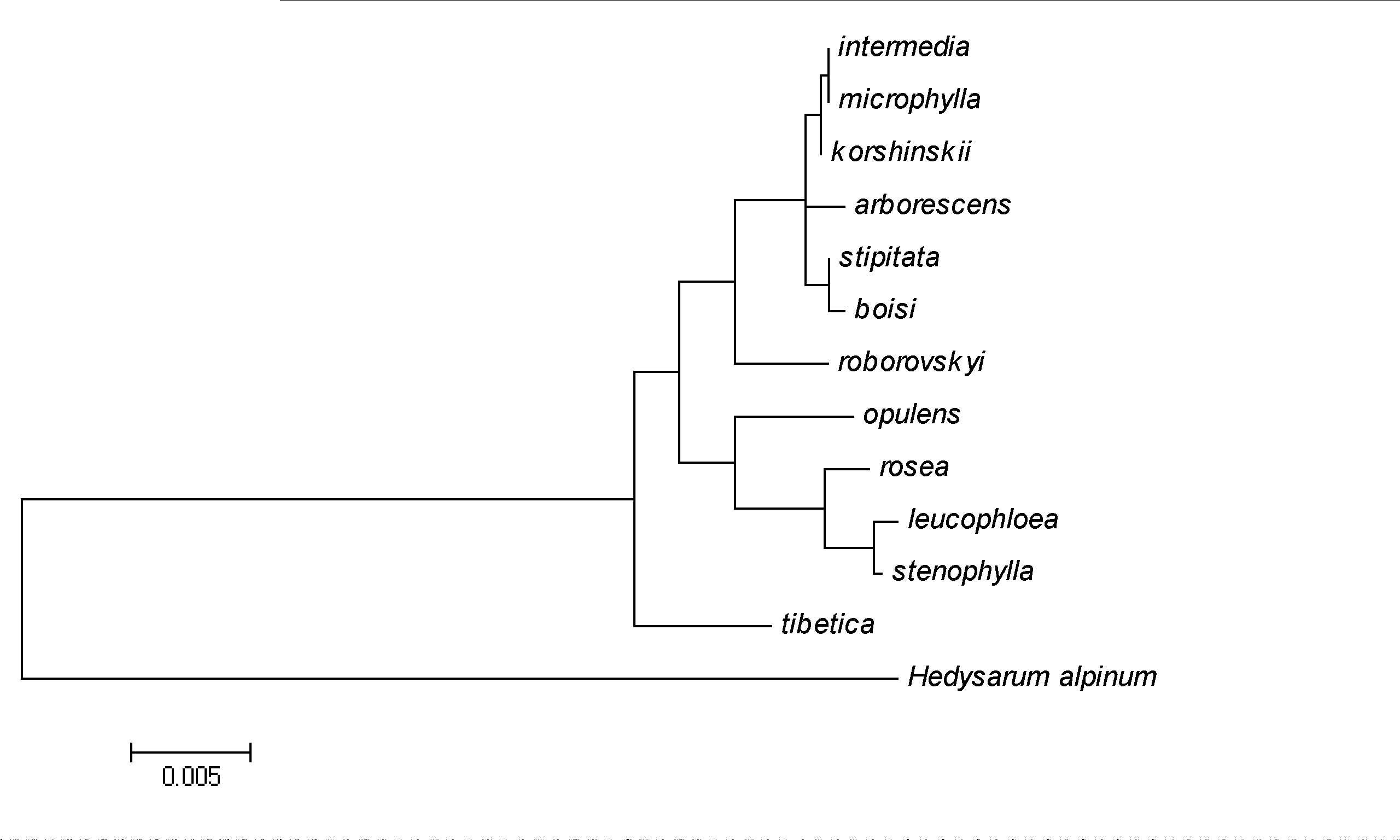


Figure S1. The phylogenetic tree of twelve *Caragana* species constructed on the basis of the *ITS*, *rbcL* and *trnS-trnG* sequences and rooted by defining *Hedysarum alpinum* as a member of the sister group for the *Caragana* species.

**
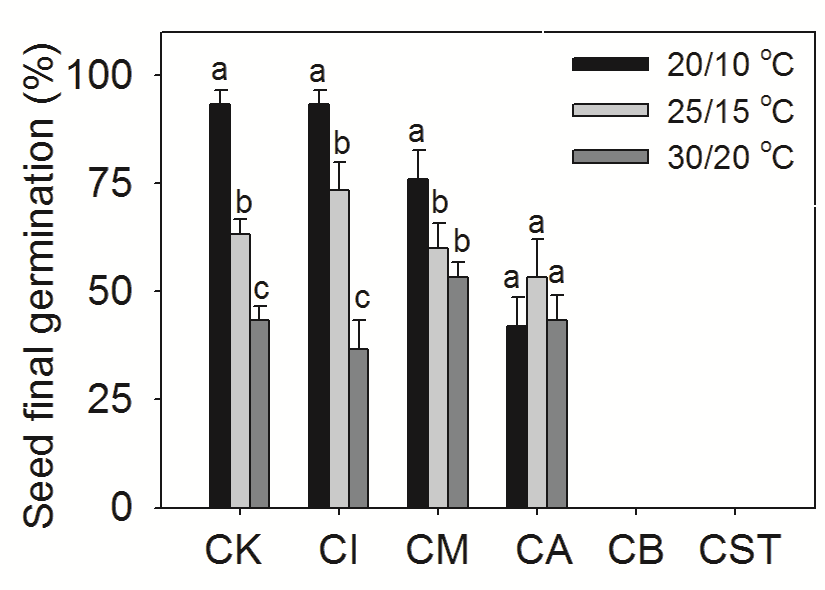
**

Figure S2. Final seed germination of six *Caragana* species from arid (*C. korshinskii,* CK), semiarid (*C. intermedia*, CI, and *C. microphylla*, CM), semihumid (*C. arborescens*, CA, and *C. boisi*, CB), and humid (*C. stipitata*, CST) habitats incubated at alternating (12/12 h) temperatures of 20/10 °C, 25/15 °C and 30/20 °C. Values were obtained from the fitted values of the data in Fig. 2. Treatment groups in each species with a different letter are significantly different from each other (*P* <0.05). Data are means + one SE of the mean (*n* = 5).

**
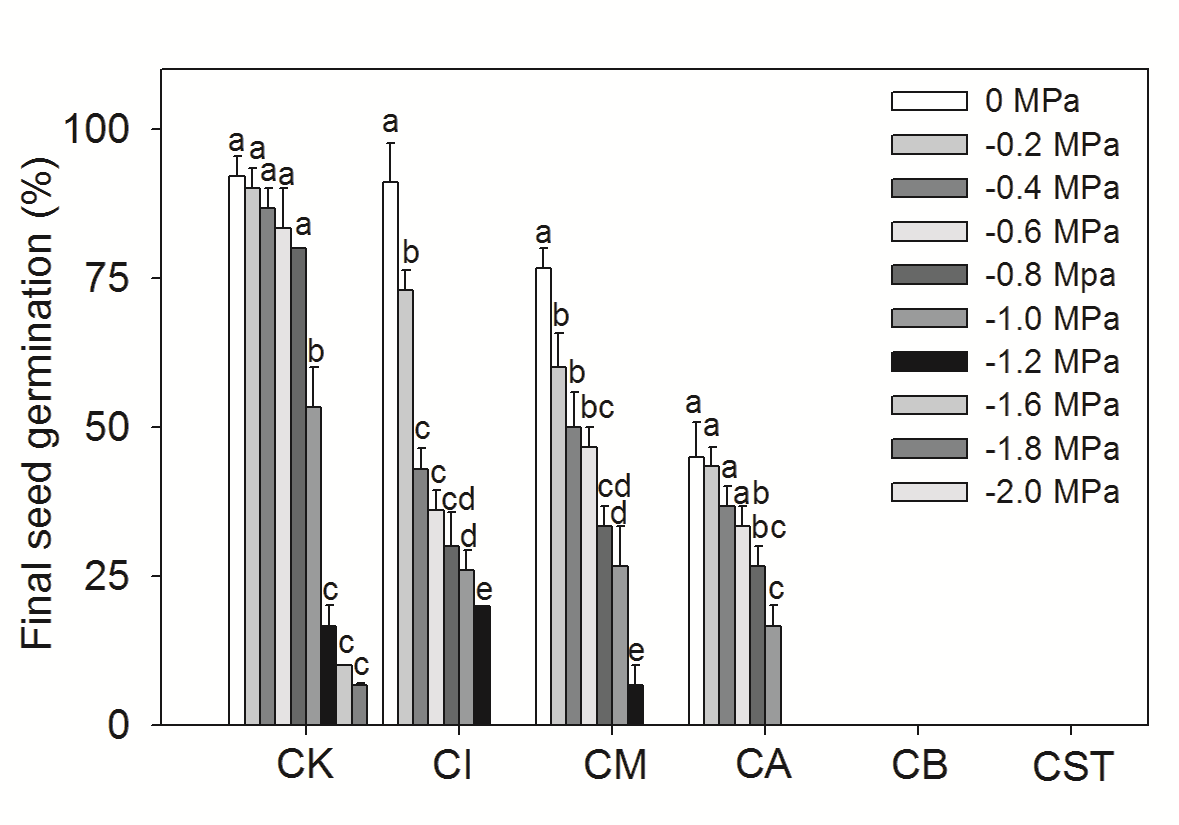
**

Figure S3. Final seed germination of six *Caragana* species from arid (*C. korshinskii*, CK), semiarid (*C. intermedia*, CI, and *C. microphylla*, CM), semihumid (*C. arborescens*, CA, and *C. boisi*, CB), and humid (*C. stipitata*, CST) habitats at water/osmotic potentials of 0.0 MPa, −0.2 MPa, −0.4 MPa, −0.6 MPa, −0.8 MPa, -1.0 MPa, −1.2 MPa, −1.6 MPa, −1.8 MPa and −2.0 MPa at 20/10 °C. Values were obtained from the fitted values of the data in Fig 2. Treatment groups in each species with a different letter are significantly different from each other (*P* <0.05). Data are means + one SE of the mean (*n* = 5).

**
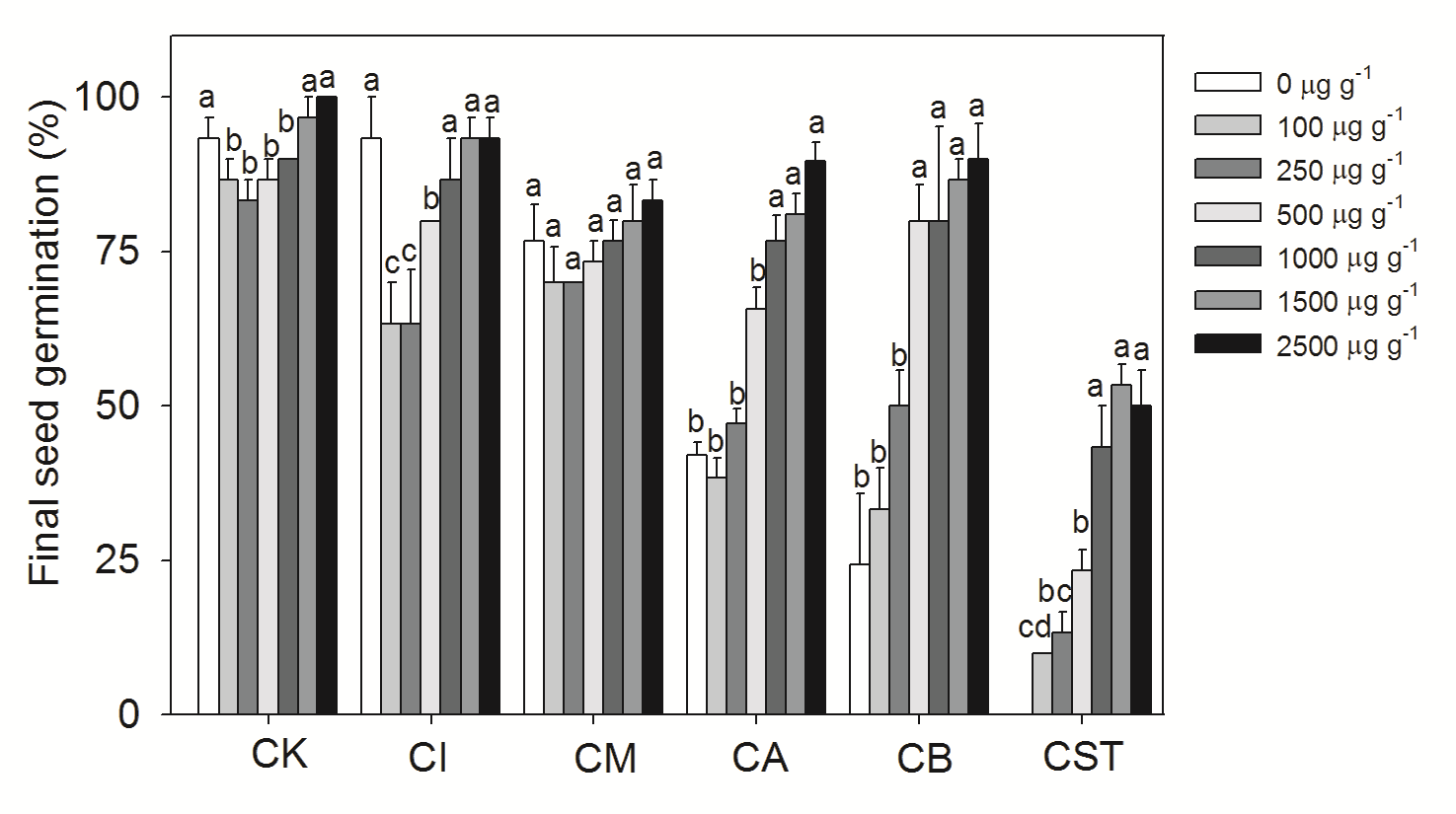
**

Figure S4. Final seed germination of six C*aragana* species from arid (*C. korshinskii*, CK), semiarid, (*C. intermedia*, CI, and *C. microphylla*, CM), semihumid (*C. arborescens*, CA, and *C. boisi*, CB), and humid (*C. stipitata*, CST) habitats at GA_3_ solution concentrations of 0 μg g^-1^, 100 μg g^-1^, 250 μg g^-1^, 500 μg g^-1^, 1000 μg g^-1^, 1500 μg g^-1^ and 2500 μg g^-1^ at 20/10 °C. Values were obtained from the fitted values of the data in Fig. 4. Treatment groups in each species with a different letter are significantly different from each other (*P* <0.05). Data are means + one SE of the mean (*n* = 5).


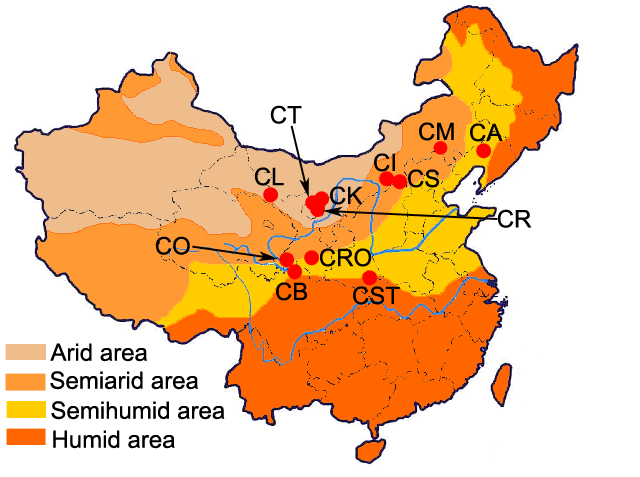


Figure S5. Map of mainland China showing where the seeds of *Caragana* were collected. *C. korshinskii* (CK), *C. roborovskyi* (CR), *C. leucophloea* (CL) and *C. tibetica* (CT) were collected in western Inner Mongolia and/or in the middle of the Hexi Corridor in Gansu Province where the annual precipitation is less than 200 mm, *C. intermedia* (CI), *C. stenophylla* (CS) and *C. microphylla* (CM) were collected in semiarid regions of middle and eastern China where the annual precipitation is between 200 mm and 400 mm, *C. opulens* (CO), *C. rosea* (CRO), *C. arborescens* (CA) and *C. boisi* (CB) were collected in the semihumid region of eastern and central China where the annual precipitation is between 400 mm and 800 mm, and *C. stipitata* (CST) was collected in the humid Hua mountains where the annual precipitation is 1000 mm. The map was created using Adobe PhotoShop CS version 8.0.1 (Adobe Systems, Inc, San Jose, California, USA).


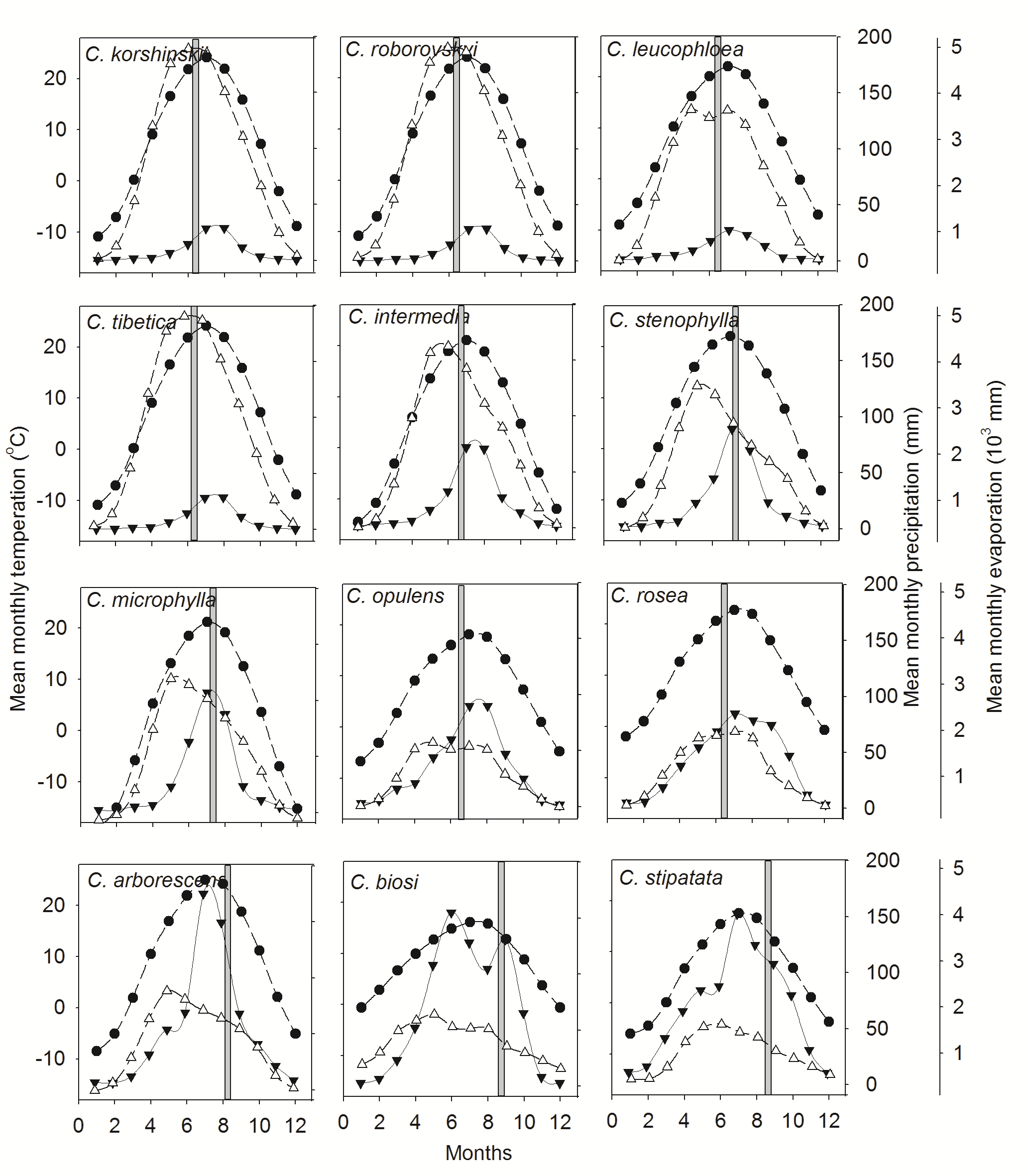


Figure S6. Mean monthly temperature (●), mean monthly precipitation (▼) and mean monthly evaporation (△) of the habitats of the 12 *Caragana* species. Shaded areas indicate the period of seed maturation in their environments, in mid-June for *C. korshinskii*, *C. roborovskyi and C. leucophloea* from arid habitats and *C. rosea* from semihumid habitats, at the end of June for *C. tibetica* from arid habitats, *C. intermedia* from semiarid habitats, and *C. opulens* from semihumid habitats, in mid-July for *C. stenophylla* and *C. microphylla* from semiarid habitats, in early August for *C. arborescens* from a semihumid habitat, and in mid-August for *C. boisi* from a semihumid habitat and *C. stipitata* from a humid habitat. Data of mean monthly temperature, mean monthly precipitation and mean monthly evaporation are averaged from 1971 to 2000 (data after 2000 not supplied) from the China Meteorological Data Sharing Service system.
